# Supplementary material for: The diagnostic and prognostic value of UBE2T in intrahepatic cholangiocarcinoma
Source: PeerJ. 2020 Jan 27;8:e8454. doi: 10.7717/peerj.8454 (PMC6991121; doi:10.7717/peerj.8454)
Supplement: Supplemental Information 1 [file peerj-08-8454-s001.docx]

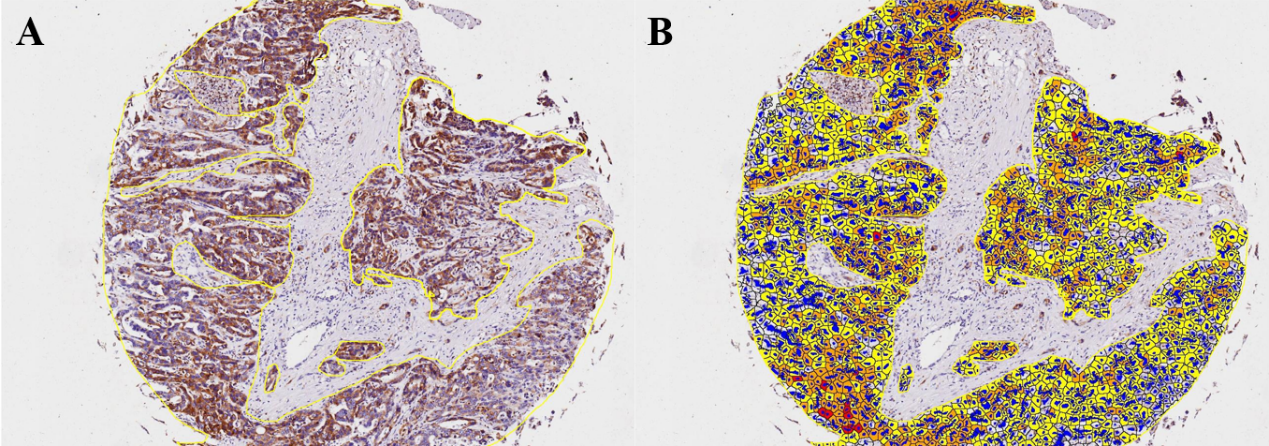


Supplementary Figure 1: The computational procedure of the HALO software for determining the H-score.


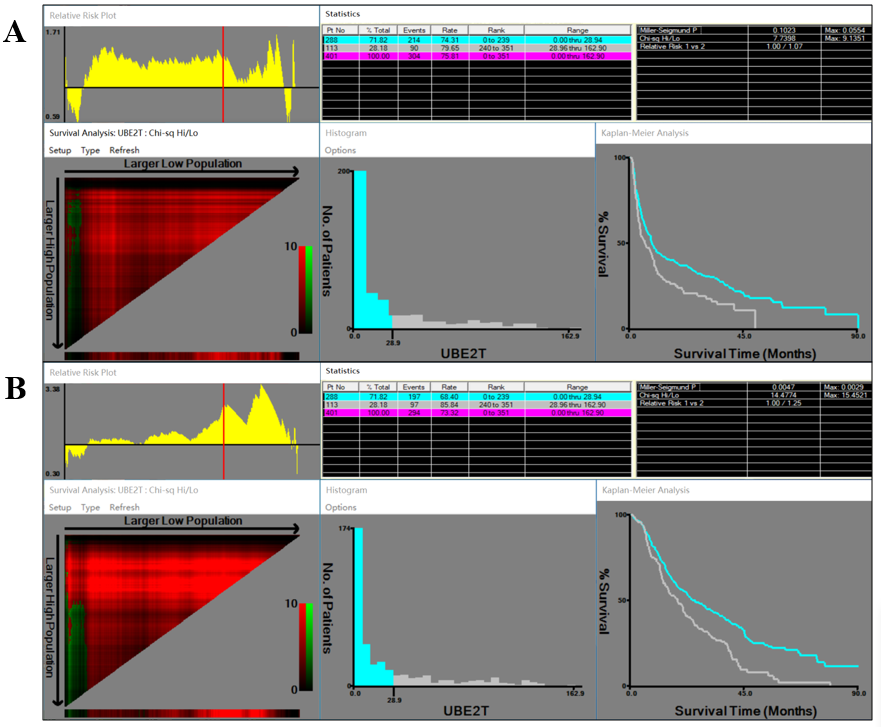


Supplementary Figure 2: X-tile analysis of prognosis based on the H-score. X-tile plots showing time to recurrence (A) and overall survival (B).

| Supplementary Table 1 Baseline characteristics of patients with IHBD (N=13), BilIN-1/2 (N=23), and BilIN-3 (N=11). | | | | |  |
| --- | --- | --- | --- | --- | --- |
| Characteristics | IHBD | BilIN-1/2 | BilIN-3 | P value | |
| Age, years | 43.61±8.98 | 56.00±11.36 | 54.82±14.38 | 0.010 | |
| Sex |  |  |  | 0.250 | |
| Male | 7(53.8%) | 6(26.1%) | 4(36.4%) |  | |
| Female | 6(46.2%) | 17(73.9%) | 7(63.6%) |  | |
| AFP, ng/mL | 61.98±201.29 | 6.68±10.79 | 28.17±86.15 | 0.378 | |
| CEA, ng/mL | 4.76±5.66 | 7.68±14.17 | 4.05±3.99 | 0.584 | |
| CA199, ng/mL | 62.77±126.62 | 119.47±281.32 | 131.79±293.36 | 0.771 | |
| TBIL, μmol/L | 18.68±15.62 | 19.84±18.08 | 29.97±61.88 | 0.652 | |
| ALB, g/L | 41.95±4.13 | 40.61±4.42 | 40.65±4.98 | 0.667 | |
| ALT, U/L | 45.68±63.81 | 38.44±30.50 | 51.58±59.99 | 0.753 | |
| GGT, U/L | 195.23±304.23 | 178.43±175.70 | 258.27±294.82 | 0.674 | |
| ALP, U/L | 93.28±102.65 | 80.94±76.41 | 178.97±117.94 | 0.022 | |
| HBsAg |  |  |  | 0.777 | |
| Positive | 4(30.8%) | 6(26.1%) | 2(18.2%) |  | |
| Negative | 9(69.2%) | 17(73.9%) | 9(81.8%) |  | |
| IHBD intrahepatic bile duct, BilIN biliary intraepithelial neoplasia, AFP alpha fetoprotein, CEA carcinoembryonic antigen, CA199 carbohydrate antigen 19-9, TBIL total bilirubin, ALB albumin, ALT alanine aminotransferase, GGT gamma glutamyltransferase, ALP alkaline phosphatase, HBsAg hepatitis B surface antigen. | | | | |  |
