# Supplementary material for: The diagnostic and prognostic value of UBE2T in intrahepatic cholangiocarcinoma
Source: PeerJ. 2020 Jan 27;8:e8454. doi: 10.7717/peerj.8454 (PMC6991121; doi:10.7717/peerj.8454)
Supplement: Supplemental Information 3 [file peerj-08-8454-s003.docx]

**Miame Checklist**

(Sections in **BOLD** represent what each researcher must record about every slide and/or experiment)

Part 1 Experiment description

*“The minimal information required in this section includes the type of the experiment (such as normal-versus-diseased comparison, time course, dose response, and so on) and the experimental variables, including parameters or conditions tested (such as time, dose, genetic variation or response to a treatment or compound).”*

*“this section specifies the experimental relationships between the array and sample entities—that is, which samples and which arrays were used in each hybridization assay. Each of these will be assigned unique identifiers that are cross-referenced with the information provided in the following sections.”*

-         **mouse type:** Not applicable

-         **experimental variables (runners vs. non-runners, high fat vs. low fat):** Not applicable

-         **n-count:** 448 cases

-         **tissues used for slide:** intrahepatic bile duct (13 cases), low-grade intraepithelial neoplasia (23 cases), high-grade intraepithelial neoplasia (11 cases), intrahepatic cholangiocarcinoma (401 cases)

-         **mouse age, and other variables (wean weight, pooled samples, etc.):** Not applicable

Part 2 Array design.

*“The aim of this section is to provide a systematic definition of all arrays used in the experiment, including the genes represented and their physical layout on the array.”*

*“The array-type definition includes information common to all arrays of a particular type (such as glass-slide spotted with PCR-amplified cDNA clones) as well as precise descriptions of the physical content of each element (spot or feature). This section consists of three parts: (i) a description of the array as a whole (such as platform type, provider and surface type); (ii) a description of each type of element or spot used (properties that are typically common to many elements, such as 'synthesized oligo-nucleotides' or 'PCR products from cDNA clones'); and (iii) a description of the specific properties of each element, such as the DNA sequence and, possibly, quality-control indicators.”*

-         **Array series:** tissue microarrays

-         **Deconvoluted spot list with gene names:** Not applicable

-         **Array type (mouse, human, cDNA, oligo, number of genes):** Human tissue microarrays

-         **Array size:** 9 rows × 10 columns, with diameter of 1.5 mm

**-         Slide type (and coating):** slides coated with 3-aminopropyltriethoxysilane

 Part 3 Samples

*“The MIAME 'sample' concept represents the biological material (or biomaterial) for which the gene expression profile is being established. This section is divided into three parts which describe the source of the original sample (such as organism taxonomy and cell type) and any biological*in vivo*or*in vitro*treatments applied, the technical extraction of the nucleic acids, and their subsequent labeling.”*

-         **Cy3/Cy5 labels for tissues:** Not applicable

-         **Dye swap? Or reference control? :** Not applicable

-         **Labelling protocol used:** Not applicable

-         **Sample extraction protocol used:** Not applicable

-         **Amount of sample labelled:** Not applicable

 Part 4 Hybridizations

*“This section defines the laboratory conditions under which the hybridizations were carried out. Other than a free-text description of the hybridization protocol, MIAME requires that a number of critical hybridization parameters are explicitly specified: choice of hybridization solution (such as salt and detergent concentrations), nature of the blocking agent, wash procedure, quantity of labeled target used, hybridization time, volume, temperature and descriptions of the hybridization instruments.”*

-         **Hybridization protocol:** Not applicable

-         **ALL modifications and deviations from the protocol:** Not applicable

-         **Manual hybridization or automatic chamber? :** Not applicable

-         **Number of slides done at the same time:** Not applicable

-         **Hyb time:** Not applicable

-         **Number of washes:** Not applicable

-         **Amount of labelled sample hybridized:** Not applicable

-         **Labelling efficiency:** Not applicable

Part 5 Measurements

*“Image data should be provided as raw scanner image files (such as TIFF), accompanied by scanning information that includes relevant scan parameters and laboratory protocols.”*

-         **Which version of scanner software used:** K-Scanner (version V1.0)

-         **Laser power for scan:** 350 watts

-         **Instrument model numbers:** KF-PRO-005-EX

-         **Must save original .tiff format images (composite image is optional)**

*For each experimental image, a microarray quantification matrix contains the complete image analysis output as directly generated by the image analysis software (normally provided as separate spreadsheet-type files). Note that for a given image this is a 2D matrix, where array elements (spots or features) constitute one dimension and quantification types (such as mean and median intensity, mean or median background intensity) are the second dimension.*

-         **Normalization protocol:** Not applicable

-         **Does the scanner software subtract background? How much?:** Not applicable

-         **Spot raw values, background intensity, ch1 and 2 intensity, etc.:** Not applicable

-         **Corresponding gene name:** Not applicable

-         **Methods of analysis (MAN, Spotfire, Genespring) be detailed.:** Not applicable

-         **Normalized to controls? Controls removed? All normalization parameters:** Not applicable

-         **Name of Images, Experiment, and location of files.:** Not applicable

-         **Lowess or other normalization if used (and parameters):** Not applicable

*Finally, the gene expression matrix (summarized information) consists of sets of gene expression levels for each sample. If microarray quantification matrices can be considered spot/image centric, then the gene expression matrix is gene/sample centric. At this point, the expression values may have been normalized, consolidated and transformed in any number of ways by the submitter in order to present the data in a form amenable to scientific analysis. Rather than attempting to impose a standard for gene expression values, MIAME indicates preferred detailed specifications of all numerical calculations applied to unprocessed quantifications in (b) that have led to the data in (c). Experimenters are encouraged, though not required, to provide reliability indicators (such as s.d.) for each data point.*

-         **Output file:** Not applicable

-         **Normalized ratios:** Not applicable

-         **Numerical manipulations:** Not applicable

-         **Cut off values:** Not applicable

Part 6 Normalization controls

*“A typical microarray experiment involves a number of hybridization assays in which the data from multiple samples are analyzed to identify relative changes in expression levels, identify differentially expressed genes and, in many cases, discover classes of genes or samples having similar patterns of expression.”*

-         **Hypothesis:** Not applicable

-         **Gene expression patterns found:** Not applicable

-         **Controls used, normalization methods used (see above) :** Not applicable
